# Treatment of anterior open bite by posterior maxillary segmental osteotomy and miniplates: a case report

**DOI:** 10.1186/s40902-020-00265-4

**Published:** 2020-06-15

**Authors:** Sung-Kwon Choi, Kyung-Hwan Kwon

**Affiliations:** 1 College of Dentistry, Graduate School of Wonkwang University, Muwanglo 895, Iksan, Jeonlabookdo Republic of Korea; 2 Department of Oral and Maxillofacial Surgery, College of Dentistry, Wonkwang University, Iksan, Republic of Korea

**Keywords:** Anterior open bite, Posterior maxillary segmental osteotomy (PMSO), Skeletal anchorages, Miniplates

## Abstract

**Background:**

Anterior open bite is a challenging malocclusion to correct orthodontic treatment. Anterior open bite associated with over-erupted posterior teeth and long lower facial height should be treated by reduction of posterior dimension for esthetic results. Although the possibility of orthodontic treatment of an anterior open bite has increased with the introduction of skeletal anchorage, there are still cases requiring surgery for various reasons.

**Case presentation:**

This case report covers an anterior open bite of a 25-year-old man successfully treated with the posterior maxillary segmental osteotomy (PMSO) and miniplates. After the pre-surgical orthodontic treatment, the PMSO between canines and first premolars was performed under local anesthesia and miniplates were placed on the zygomatic buttress. As a result of 28 months of treatment, an impaction amount of 3.5 mm was obtained in the maxillary posterior teeth, and the facial esthetics improved at rest and smile.

**Conclusion:**

The impaction of the posterior dentoalveolar segment using the PMSO can be a good treatment option in patients with anterior open bite showing long lower facial height.

## Background

Anterior open bite is a challenging malocclusion to treat orthodontically. It could result from various factors such as habits, tongue postures, airway obstruction, vertical skeletal growth problems, and temporomandibular joint disorders [1–3].

Anterior open bite is often associated with over-erupted posterior teeth and long lower face height [4, 5]. In this type of malocclusion, intrusive mechanics are required for optimal esthetic results. Before the introduction of skeletal anchorages, a reduction of the posterior vertical dimension can be achieved by orthognathic surgery. The intrusion of posterior teeth became possible by the use of skeletal anchorages. However, the treatment duration and retention of the treatment results are still issues [6–8].

Posterior maxillary segmental osteotomy (PMSO) is a surgery for repositioning of the posterior dentoalveolar segment. It has been mainly used to make an intermaxillary space for the restoration of mandibular teeth [9, 10]. In the area of orthodontics, the segmental surgery can be used to correct the transverse and vertical discrepancies of the maxilla [11, 12]. The PMSO has advantages than orthodontic intrusion when there are many teeth to be intruded or when a large amount of intrusion is required.

This case report covers an anterior open bite of a 25-year-old man successfully treated with the PMSO and miniplates.

## Case presentation

**Fig. 1:**
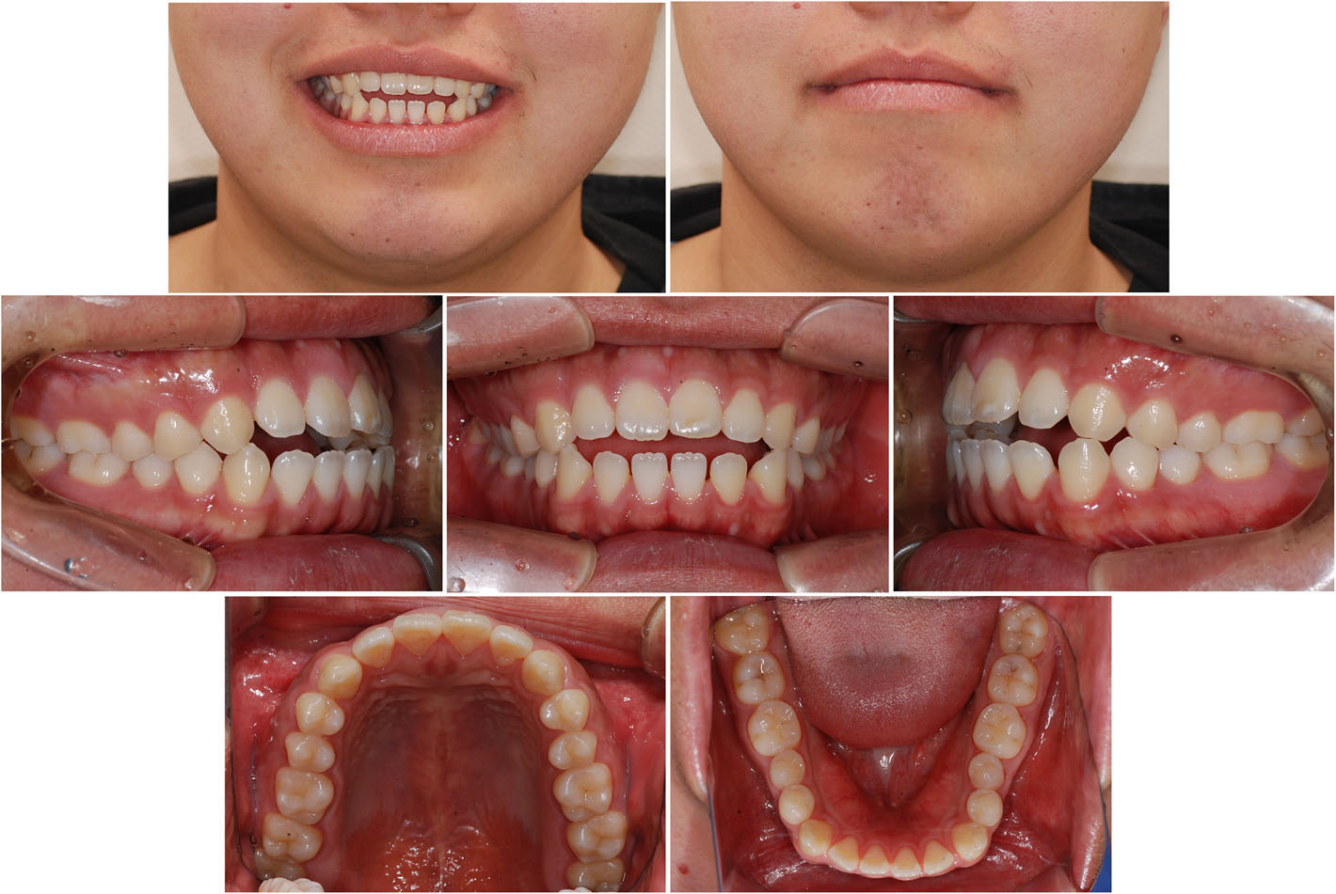
Pre-treatment photographs

A 25-year-old male patient visited our Department of Orthodontics with a chief complaint of anterior open bite. Clinical examination revealed a class I molar and canine relationship. The overjet and overbite were 1 mm and -2mm, respectively, and open bite was limited only to the incisors. The exposure of maxillary anterior teeth in a smile was adequate, and lip competence could be achieved with some mentalis strain. Reverse smile arc and excessive exposure of mandibular teeth were observed in facial photographs (Fig. 1). The cephalometric analysis showed a skeletal class II relationship with a prognathic maxilla, normovergent pattern, and labioversion of maxillary and mandibular incisors (details are shown in Table 1). All third molars were fully erupted and well occluded.

**Table 1:** Cephalometric measurements

Measurement	Pre-treatment	Post-treatment	Post-retention (49 m)
SNA	88.0	88.4	88.3
SNB	82.9	83.8	84.0
ANB	5.1	4.6	4.3
Wits	− 1.8	− 1.3	− 1.4
SN-MP	32.3	30.7	30.8
U1 to SN	118.9	118.1	119.3
IMPA	100.9	96.5	97.4
Interincisal angle	107.9	114.6	112.6
Upper lip E-plane	− 0.3	− 0.8	− 1.8
Lower lip E-plane	5.1	4.2	4.1
U1-NF	37.8	38.8	38.8
U6-NF	30.5	27.0	27.4

The patient was diagnosed as skeletal class II and dental class I with normovergent pattern, anterior open bite, and proclination of the upper and lower incisors.

The first treatment option was a segmental Le Fort I osteotomy. This procedure can immediately reduce the large amount of posterior vertical dimension and correct two occlusal planes. Since the patient was unable to be hospitalized, he rejected orthognathic surgery under general anesthesia.

The second treatment option was orthodontic intrusion of maxillary teeth using a skeletal anchorage system. A large amount of intrusion of all maxillary teeth except central and lateral incisors was needed for optimal esthetic results in this patient. We thought that miniplates were more suitable than mini-implants because miniplates can be placed apically enough and provide stable intrusive forces. Along with miniplate placement, the supplementary surgery was considered to reduce the treatment time. A surgical impaction of posterior segments had the advantage of shortening the treatment time and retention of treatment results. Since the patient wanted treatment to end quickly, he agreed to this plan with PMSO and informed consent was obtained.

A fixed tongue crib was delivered to control tongue posture. 0.018-inch standard edgewise brackets were bonded, and 0.0175-inch twisted stainless steel wires were inserted in both arches. Brackets on maxillary canines and premolars were bonded to specially designed position to widen the inter-radicular space, and step bends were added between maxillary canine and premolar to prevent extrusion of anterior teeth.

Four months later, the wires were changed to 0.016-inch stainless steel wires, and open coil springs were inserted between maxillary canines and premolars. After 12 months of alignment, 0.016 × 0.022-inch sectional stainless steel wire was inserted in the maxillary arch, and the patient was referred to the Department of Oral and Maxillofacial Surgery for the PMSO.

**Fig. 2:**
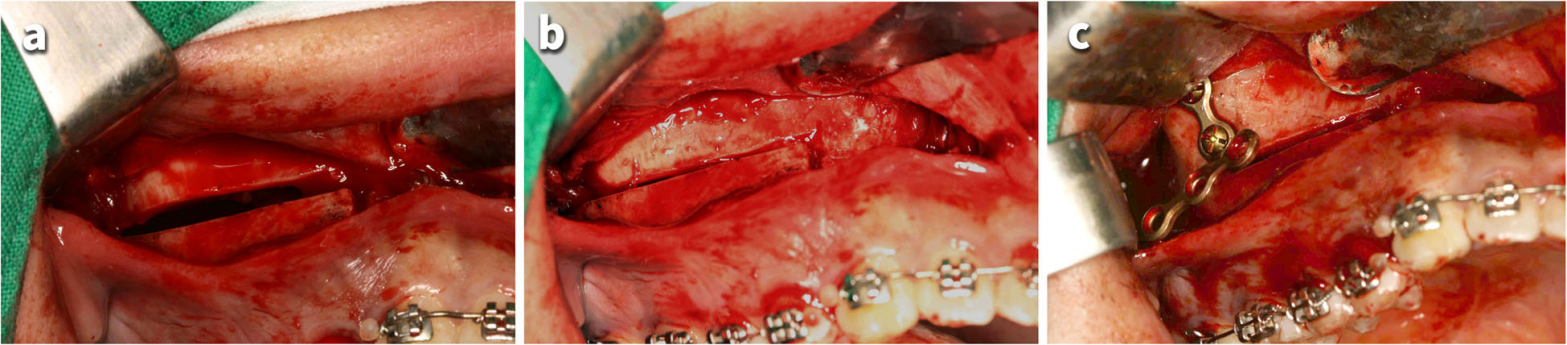
Intraoperative photographs of posterior maxillary segmental osteotomy (PMSO) and miniplate placement. **a** Three-mm-wide buccal cortical bone was removed from the horizontal osteotomy line. **b** The posterior segment was impacted after the palatal bone cut by curved osteotome. **c** The miniplate placed on the zygomatic buttress area

The surgery was performed under local anesthesia by dividing the left and right sides in consideration of dining convenience (Fig. 2). After full-thickness mucoperiosteal flap was elevated, buccal bone cutting was initiated from inter-radicular space between canine and first premolar, taking care not to damage the root surface. The horizontal cutting was extended to pterygopalatine junction at 5 mm above the apices of molars. A bone fragment of 3 mm in width was removed from the horizontal osteotomy line for posterior impaction. The palatal bone was cut by curved osteotome without flap elevation to maintain the blood supply. The bone segment was moved apicolaterally, and T-shaped miniplate was placed on the zygomatic buttress. Posterior teeth were ligated with 0.012-inch dead soft wire to miniplates to hold the bone segment on the place. After surgery, antibiotics and non-steroidal anti-inflammatory drugs were prescribed.

**Fig. 3:**
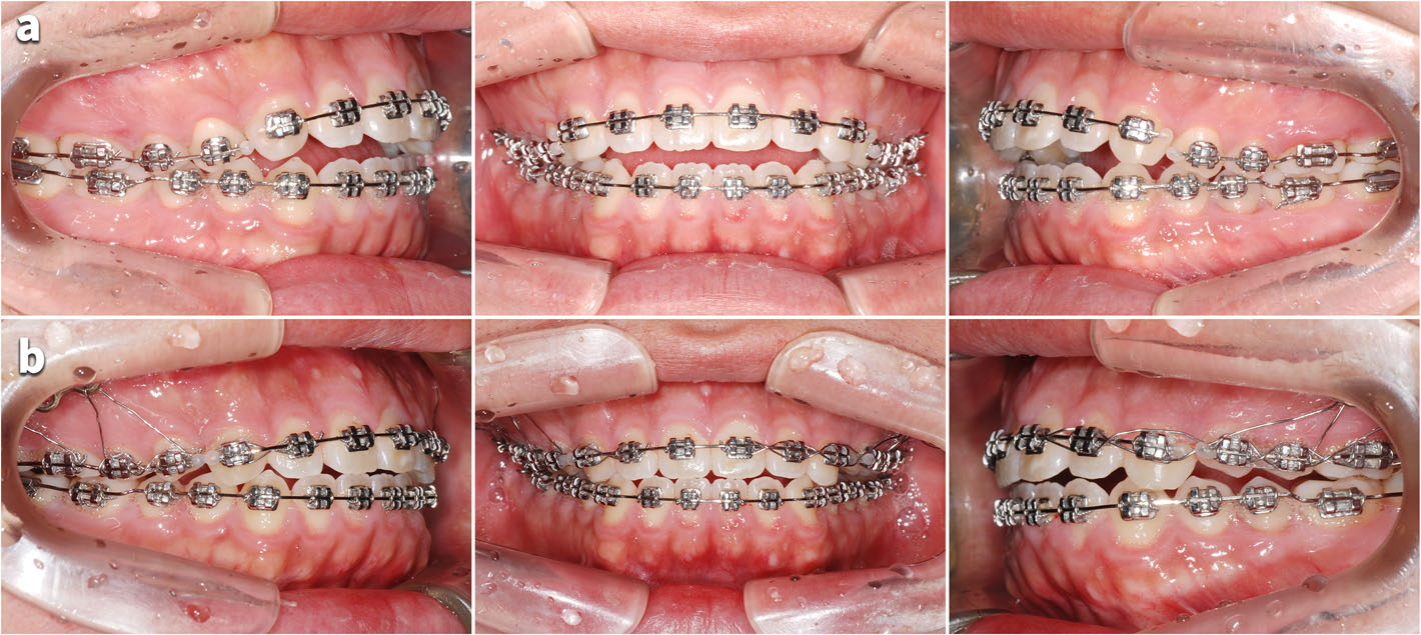
Occlusal changes after the PMSO. **a** Before the PMSO. **b** After the PMSO

**Fig. 4:**
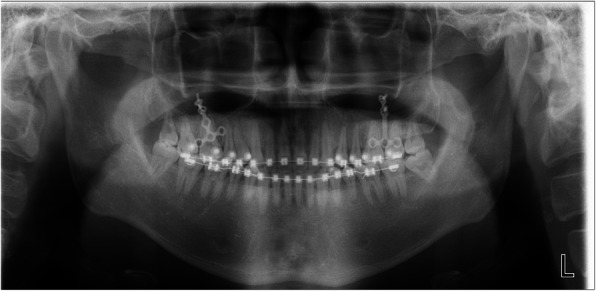
Post-surgical panoramic radiograph

The patient was followed up 2 days after the surgery. Moderate facial swelling and ecchymosis on his maxillary area were identified, but they were relieved after medication and wound irrigation of 2 weeks. A second surgery was performed at the opposite side after a month from the first surgery. The anterior open bite improved immediately after the surgery (Figs. 3 and 4).

A month later from the second surgery, the brackets on maxillary canine were repositioned to normal position, and 0.016-inch nickel-titanium archwire was placed in the maxillary arch. A mini-implant was placed at the center of the mid-palatal suture for further intrusion of posterior teeth. Elastic threads were applied from the lingual button on posterior teeth to mid-palatal mini-implant.

The additional intrusion of posterior teeth was continued for 9 months after the surgery. The maxillary right miniplate was loosened after 6 months from the first surgery, so we replaced it with a new miniplate. The archwires were subsequently changed to 0.014-inch, 0.016-inch, and 0.016 × 0.022-inch stainless steel wire in this period. During the finishing stage, the torque of the left maxillary canine and dental midline was corrected.

**Fig. 5:**
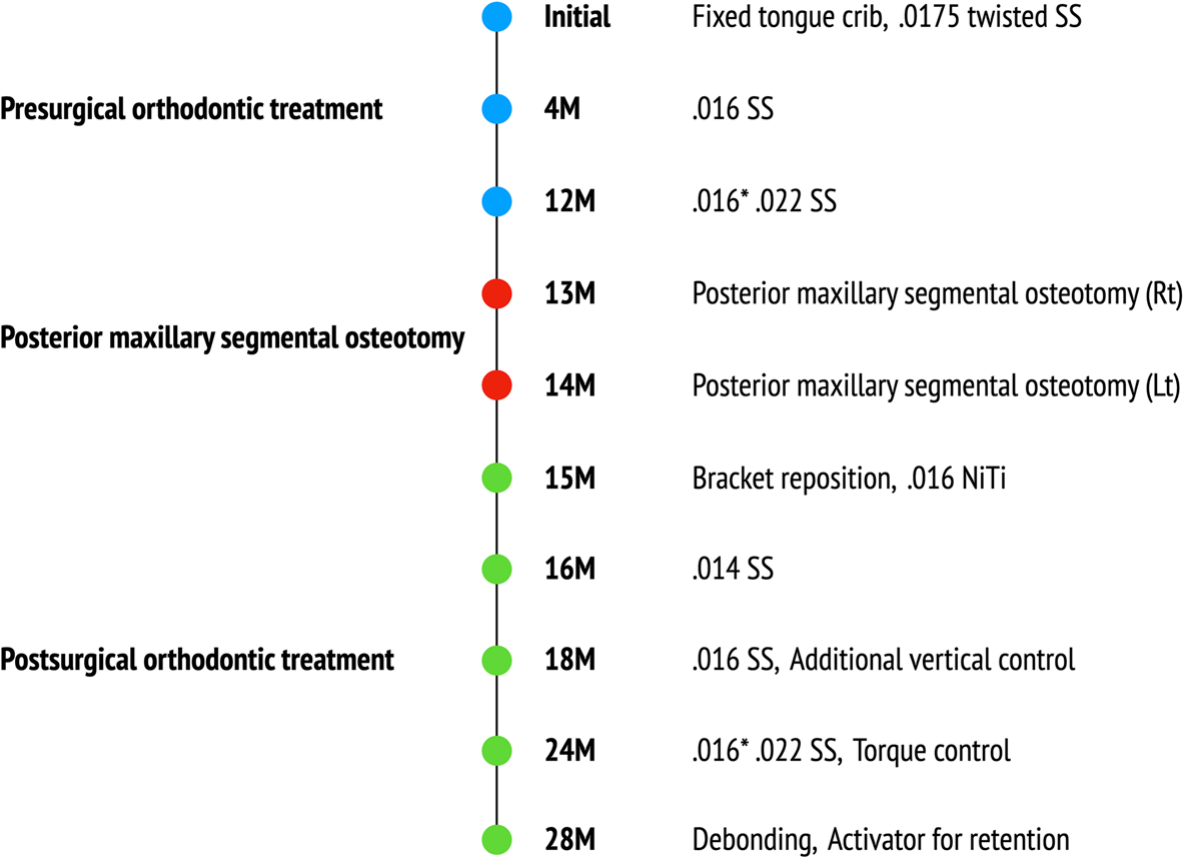
Schematic flow of treatment

After 28 months of treatment, a favorable occlusion was obtained, and orthodontic appliances were removed. Fixed retainers were bonded on both arches, and the activator was delivered for retention. A schematic flow of the treatment is shown in Fig. 5.

## Treatment results

Tongue habit was gradually improved with the use of tongue crib and was not recognized after the PMSO surgery.

**Fig. 6:**
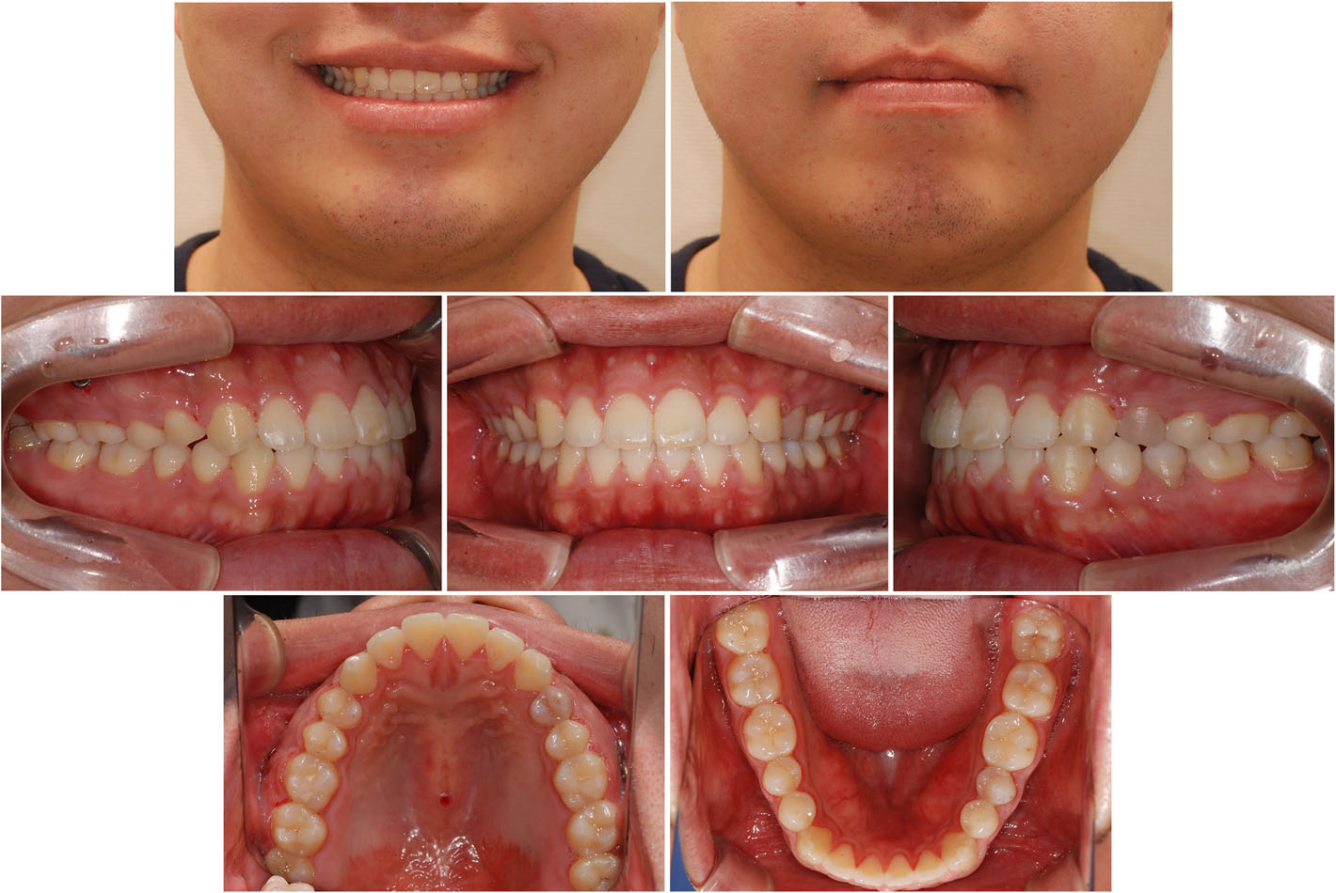
Post-treatment photographs

Facial photographs showed the improvement of the patient’s smile esthetics. Reverse smile arc and reverse lip line were corrected to a consonant smile arc and straight lip line, respectively. The exposure of lower teeth in a smile was decreased, and lip competence was achieved without mentalis strain (Fig. 6).

A post-treatment panoramic radiograph showed good root parallelism, and only mild blunting of the apices of the maxillary incisors was seen in the periapical radiograph.

**Fig. 7:**
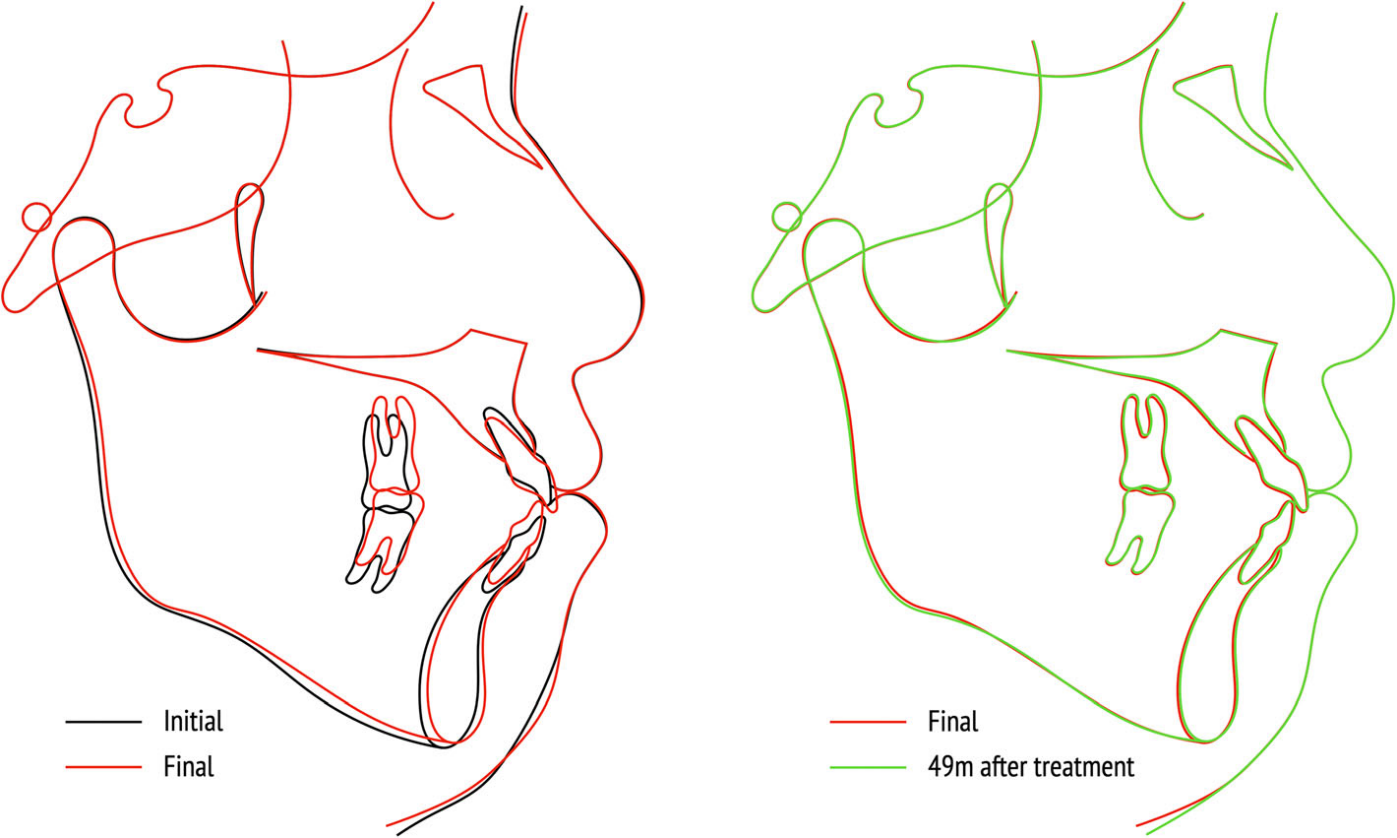
Superimposition of the cephalometric tracing. Black line, before treatment; red line, after treatment (28 months after initiation of orthodontic treatment); green line, retention after 49 months

The cephalometric superimposition showed a counterclockwise rotation of the mandible; the SN-MP angle was reduced from 32.3 to 30.7. Single occlusal plane was achieved by 3.5 mm of the intrusion of the maxillary molars and 1.0 mm of the extrusion of maxillary incisors. The mandibular molars were extruded by 1.0 mm. At the 49-month retention, treatment results were stable without opening of mandibular plane angle (Fig. 7, Table 1).

## Discussion

The introduction of skeletal anchorages has opened a new ground for the treatment of anterior open bite. Intrusion of the posterior teeth using skeletal anchorages provides more stable and esthetic results than extrusion of anterior teeth. However, the intrusion is one of the slowest movements among the various tooth movements. Therefore, the treatment duration of anterior open bite is mainly dependent on the rate of molar intrusion.

Various methods for the PMSO have been introduced according to the number of operations and the position of the osteotomy [11–13]. In this case, we followed the technique of Tuncer et al. This surgical method has the advantage of maintaining the palatal blood flow by not elevating the palatal flap. In addition, we impacted the posterior segments after removing the buccal cortical bone of 3 mm in width. To secure sufficient inter-radicular space, premolar extraction was also considered, but was dismissed because no posterior movement of the incisor was required.

Pulp necrosis occurred without apparent root damage on the adjacent tooth to the osteotomy line. Pink discoloration of right maxillary first premolar was recognized 8 months from the surgery. At that time, the patient did not have any symptoms. After 14 months, however, he complained of pain during occlusion and tenderness to percussion on this tooth. It was diagnosed as pulp necrosis, and root canal treatment was performed. Many retrospective studies reported the prevalence of the pulp necrosis after Le Fort I osteotomy ranging 0.5 to 3.4% [14–17]. Lownie et al. reported that ischemic stress induced by surgical procedures could result in inflammatory changes in pulp tissue, and there were degenerative changes with vacuolization and atrophy of the odontoblastic layer [18]. The study using laser Doppler flowmetry showed that the segmental Le Fort I surgery induced a decrease of pulpal blood flow in adjacent to vertical osteotomy line [19]. In this case, osteotomy performed on a narrow inter-radicular space appears to have affected the pulpal blood supply.

**Fig. 8:**
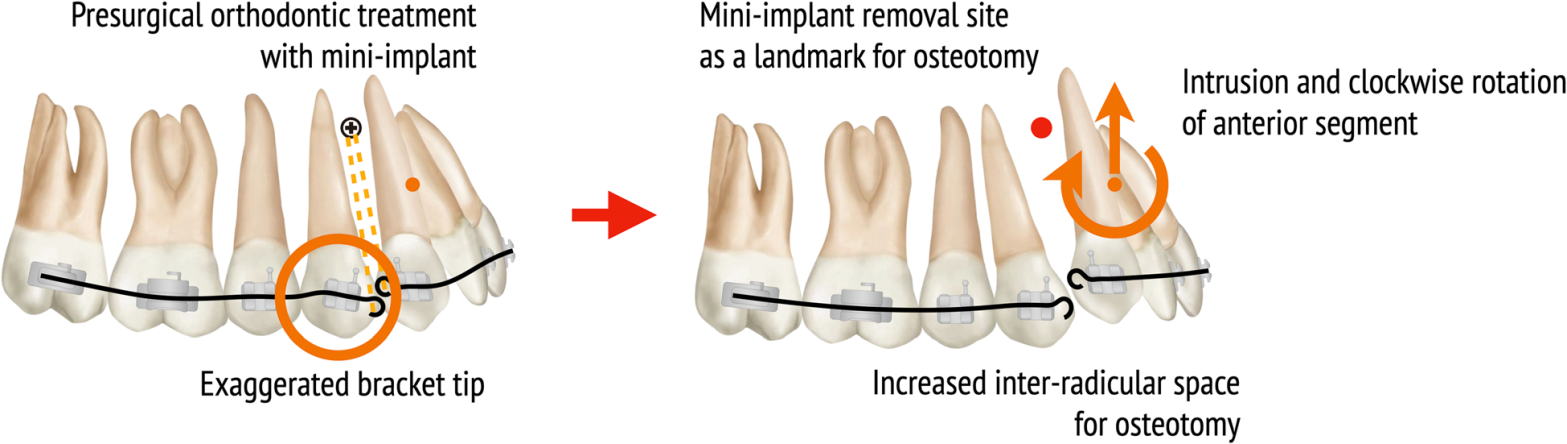
Orthodontic strategy to avoid root damage during PMSO

A sufficient impaction of posterior teeth was achieved by PMSO surgery, but the premature contacts on the canines prevented further closure of the mandible (Fig. 3). Considerable time was spent for intruding canines after PMSO surgery, and small amount of extrusion of mandibular posterior teeth occurred during this time. If we had actively intruded the maxillary canines using mini-implants before the PMSO surgery, post-surgical orthodontic treatment would have been shortened. To reduce the risk of root damage and shorten the treatment period, it is helpful to actively intrude the anterior segment by using a mini-implant and to improve the axis of the canine and premolar in pre-surgical orthodontic treatment (Fig. 8).

In this case, since the patient mainly had vertical problems including anterior open bite and long lower facial height, the impaction of the posterior teeth was the main focus in the treatment plan. The impaction of the ` through PMSO was judged to be advantageous compared to orthodontic intrusion because all the teeth except the incisors were occluded. Functional occlusion and improvement of facial esthetics were obtained through orthodontic treatment including PMSO, and this treatment results have been maintained over 4 years.

## Conclusion

The impaction of the posterior dentoalveolar segment using PMSO can be a good treatment option in patients with anterior open bite showing two occlusal planes. In order to reduce side effects and increase treatment efficiency, planned orthodontic movement must be performed in the pre-surgical orthodontic treatment, as with orthognathic surgery.

## Data Availability

Not applicable (data sharing not applicable to this article as no datasets were generated or analyzed during the current study).
